# Macrolide Treatment Failure due to Drug–Drug Interactions: Real-World Evidence to Evaluate a Pharmacological Hypothesis

**DOI:** 10.3390/pharmaceutics14040704

**Published:** 2022-03-25

**Authors:** Brian Cicali, Stephan Schmidt, Markus Zeitlinger, Joshua D. Brown

**Affiliations:** 1 Center for Pharmacometrics and Systems Pharmacology, Department of Pharmaceutics, College of Pharmacy, University of Florida, Orlando, FL 32827, USA; bcicali@ufl.edu (B.C.); sschmidt@cop.ufl.edu (S.S.); 2 Department of Clinical Pharmacology, Medical University of Vienna, 1090 Vienna, Austria; markus.zeitlinger@meduniwien.ac.at; 3 Center for Drug Evaluation and Safety, Department of Pharmaceutical Outcomes & Policy, University of Florida College of Pharmacy, Gainesville, FL 32601, USA

**Keywords:** macrolide antibiotics, treatment failure, drug–drug interactions, pharmacoepidemiology

## Abstract

Macrolide antibiotics have received criticism concerning their use and risk of treatment failure. Nevertheless, they are an important class of antibiotics and are frequently used in clinical practice for treating a variety of infections. This study sought to utilize pharmacoepidemiology methods and pharmacology principles to estimate the risk of macrolide treatment failure and quantify the influence of their pharmacokinetics on the risk of treatment failure, using clinically reported drug–drug interaction data. Using a large, commercial claims database (2006–2015), inclusion and exclusion criteria were applied to create a cohort of patients who received a macrolide for three common acute infections. Furthermore, an additional analysis examining only bacterial pneumonia events treated with macrolides was conducted. These criteria were formulated specifically to ensure treatment failure would not be expected nor influenced by intrinsic or extrinsic factors. Treatment failure rates were 6% within the common acute infections and 8% in the bacterial pneumonia populations. Regression results indicated that macrolide AUC changes greater than 50% had a significant effect on treatment failure risk, particularly for azithromycin. In fact, our results show that decreased or increased exposure change can influence failure risk, by 35% or 12%, respectively, for the acute infection scenarios. The bacterial pneumonia results were less significant with respect to the regression analyses. This integration of pharmacoepidemiology and clinical pharmacology provides a framework for utilizing real-world data to provide insight into pharmacokinetic mechanisms and support future study development related to antibiotic treatments.

## 1. Introduction

Antibiotic-resistant bacteria and the failure of antibiotic treatment are major healthcare concerns of the modern world [1]. Macrolide antibiotics were once considered first-line antibiotics against a variety of infections, such as acute respiratory infections, acute otitis media and acute sinusitis, among others. However, significant increases in treatment failure with macrolides have been observed over the past decade with reports of up to 45% in some regions of the United States, primarily due to complications such as resistance development [2,3]. In fact, due to this risk of treatment-resistant bacteria, macrolides have been removed from multiple clinical guidelines [4,5,6]. However, macrolides remain to play a critical role in the treatment of multiple infections, including community-acquired pneumonia, nontuberculosis mycobacterium, some sexually transmitted diseases and have even been explored for COVID-19 [7].

In addition to the bacterial resistance, macrolide use has been limited by adverse drug events. In fact, telithromycin, a ketolide derivative intended to overcome macrolide-related resistance mechanisms, was removed from the US market due to cardiotoxicity issues [8]. Given the clinical importance of this class of antibiotics, there is a need for innovative methods to better understand the pharmacology of macrolide antibiotics and how clinical use can be optimized for the minimization of treatment failure in real-world applications.

Clinical macrolide antibiotic treatment failure is typically associated with poor drug adherence, drug absorption or antibacterial resistance [9]. Recently, attention has increased on characterizing the influence of macrolide pharmacokinetic/pharmacodynamic (PK/PD) properties on treatment failure and the emergence of resistance [10]. It has been shown that the length of macrolide sub-minimum inhibitory concentrations (MIC) contributes to the rate of resistance mutations [11]. This finding resulted in an increased interest in better understanding the PK mechanisms associated with macrolide elimination, particularly terminal half-life, where influences on prolonged sub-MIC periods would be expected. It is important to note that the physiochemical properties of erythromycin, clarithromycin and azithromycin are substantially different, which causes a wide range of observed half-lives, terminal clearance and drug–drug interaction (DDI) risk potential. 

For example, pharmacokinetic modeling efforts have indicated that low-pH compartment “trapping” of azithromycin is at least partially responsible for its long terminal half-life and thus extended sub-MIC periods, which is not as prevalent following clarithromycin or erythromycin use [12]. This longer terminal half-life supports the findings of a double-blinded, randomized clinical trial, which showed the proportion of macrolide failure due to resistant bacteria was higher in the azithromycin arm compared to the clarithromycin or placebo arms [13]. Further, a review of antibiotic PK literature found that macrolide exposure changes can occur in the presence of DDIs, potentially influencing treatment failure [14]. Sufficiently understanding the influence of macrolide exposure, and changes of that exposure, on macrolide treatment failure is consequently important for minimizing risk of treatment resistance relative to susceptibility of the organism. 

The use of big-data analytics and real-world outcomes data for hypothesis generation, hypothesis testing and knowledge-gap identification in clinical pharmacology has been recently highlighted as a great opportunity for pharmaceutical investigations [15,16]. In fact, examples of cross-disciplinary collaborations between pharmacoepidemiologists and (quantitative) clinical pharmacologists to better evaluate mechanistic causality on real-world health outcomes have already begun to appear in the literature [17,18]. While there are pharmacoepidemiologic studies examining antibiotic use and the prevalence of antibiotic resistance, a method of combining clinical pharmacology principles with these techniques has yet to be explored for macrolide antibiotics [19]. Therefore, the aim of this work was to develop and implement a novel pharmacoepidemiologic approach for a clinical pharmacology project to (1) characterize the rate of macrolide antibiotic treatment failure within a large commercial patient population and (2) quantify the influence of drug interactions on macrolide exposure and treatment failure rates.

## 2. Materials and Methods

### 2.1. Study Design and IRB

This project utilized a retrospective, observational nested case–control approach with incidence density sampling to reach its conclusions. The database used for the analyses is certified as deidentified, and the present study was approved as exempt by the Institutional Review Board at the University of Florida (IRB201701362).

### 2.2. Data Source and Software

The data used in this project came from the IBM MarketScan Commercial Claims Databases (2006–2015). This database includes information on inpatient and outpatient medical encounters and pharmacy dispensing claims for a commercially insured patient population in the United States. Assessments of medical conditions were based on the International Classification of Diseases, 9th revision, Clinical Modification Codes (ICD-9-CM) and macrolide exposures assessed via National Drug Codes (NDCs). The beneficiaries have encrypted identifiers in the database, which allows for longitudinal follow-up. All computer code for this project’s data management and statistical analyses was written in SAS (v9.4). Additional analyses and visualizations were performed with R (v3.6) and Microsoft Excel.

### 2.3. Study Cohorts

#### 2.3.1. Common Acute Infections

To develop an appropriate dataset for this study, the following inclusion and exclusion criteria were applied. The overall goal of these criteria was to formulate a dataset containing only healthy individuals diagnosed with a common, acute bacterial infection, wherein macrolide treatment failure would not be expected nor influenced by intrinsic or extrinsic comorbidities.

##### Inclusion Criteria

Patients were initially included if *all* the following criteria were satisfied:

- Existence of a primary or secondary diagnosis code for acute bronchitis (ICD-9-CM 466.0), suppurative acute otitis media (AOM) (ICD-9-CM 382.01) or acute sinusitis (ICD-9-CM 461.9).
- Filled a prescription for a macrolide (erythromycin, azithromycin, or clarithromycin) within 5 days of infection diagnosis.
- Had longitudinal data available 12 months prior to and 60 days after infection diagnosis.

##### Exclusion Criteria

We sought to exclude patients who had prior infections, antibiotic treatment, or who were immunocompromised to rule-out prior treatment failure or high-risk groups. Thus, patients were dropped from the initially included population if *any* of the following criteria were satisfied:

- The patient was hospitalized within 30 days prior to the inclusion date.
- The patient had any of the inclusion criteria infections (ICD-9-CM 466.0, ICD-9-CM 382.01, or ICD-9-CM 461.9) within 6 months prior to the inclusion date.
- The patient had a previous pharmacy claim for an antibiotic within 30 days prior to the diagnosis date.
- The patient had an immunocompromising disorder within 2 years prior to the inclusion date. Disorders included inherited immune/autoimmune disorders, HIV/AIDs, any form of cancer or any type of organ transplant.

#### 2.3.2. Bacterial-Specific Community-Acquired Pneumonia (CAP)

To develop the dataset for bacteria-specific infections, the same inclusion and exclusion criteria for the acute infection analysis were utilized with the exception of the diagnosis coding, which was adjusted within the inclusion criteria. Specifically, bacteria-specific CAP ICD-9-CM codes were used instead of the ICD-9-CM codes for the common infections of interest. A complete list of these ICD-9-CM codes can be found in Table S1 of the Supplementary Materials.

#### 2.3.3. Case–Control Identification

The study used a nested case–control design. This design encompasses a typical case–control approach, but the cases and controls are “nested” within a specific cohort. Here, that cohort was identified by the above inclusion and exclusion criteria and is a cohort of people with an infection treated with a macrolide. From these, the identification of cases (macrolide treatment failure) and controls (macrolide treatment success) was established using previously defined and validated methodologies for both the macrolides and infections of interest [20,21,22]. Specifically, macrolide treatment failure was determined by the existence of any one or more of the following scenarios within a patient’s claims data:

- A refill of the index drug (original macrolide) within 30 days of the initial dispense date.
- A fill of another antibiotic within 30 days of initial dispense date.
- A hospitalization or emergency department visit within 30 days of inclusion date due to the same diagnostic code as the original infection.

#### 2.3.4. Case–Control Matching

To ensure results of the statistical analyses were unbiased, a matching methodology was conducted to create the final case–control pairs. Case–control pairs were exact matched 1:1, without replacement, on the following parameters: age, sex, year of infection, number of medications, macrolide received, dose of the macrolide and Elixhauser comorbidity score [23]. Note that for the bacteria-specific CAP analysis, control replacement was allowed, and matches on age, number of medications and Elixhauser score were allowed to vary by a maximum of 2 units rather than exact matched to increase sample size without compromising matching integrity.

### 2.4. Pharmacokinetic Parameters of the Analysis

To quantify the role of macrolide pharmacokinetics on macrolide treatment failure, covariates based on drug exposure changes were leveraged. Specifically, literature-reported in vivo drug interaction studies in which macrolide exposure was altered were used to identify DDI pairs. In general, all three macrolides of interest are hepatically cleared, with erythromycin and clarithromycin metabolism predominantly attributed to CYP3A4 and azithromycin predominantly associated with unchanged biliary excretion [24,25]. 

To identify drug interactions that have shown significant in vivo changes in macrolide exposure, the University of Washington’s Drug Interaction Database (UWDIDB) was utilized. The UWDIDB is a web-based tool that integrates information from peer-reviewed literature, public repositories, textbooks, approved prescribing information and new drug approval packages into a format that can be queried for analysis and research questions, including the extent of exposure change within in vivo drug interaction studies [26]. Using this resource, all in vivo DDIs which altered the systemic exposure of a macrolide, measured via change in total area under the plasma concentration–time curve (AUC), were identified for the macrolides of interest. These compiled macrolide–DDI perpetrator pairs were then encoded into the data analysis script, and patients taking both the perpetrator and macrolide victim concomitantly were identified. This identification allowed for the flagging of patients who were most likely experiencing a DDI on their respective macrolide treatment, thus allowing the assumption that their macrolide exposure was altered based on the clinical DDI reports obtained from the UWDIDB. 

The following DDI flags were generated for this study: No Interaction (≤25% increase or decrease in AUC), Mild Induction DDI (50% > AUC decrease > 25%), Moderate Induction DDI (>50% AUC decrease), Mild Inhibitor DDI (50% > AUC increase > 25%) and Moderate Inhibitor DDI (>50% AUC increase). It is noteworthy that while the induction classifications are in line with the U.S. Food and Drug Administration and the European Medicines Agency guidance definitions, the inhibition classifications are not [27,28]. According to both guidances, a moderate inhibitor DDI is one that has a >200% AUC increase, thereby resulting in all inhibitor interactions within this project being classified as either no interaction or mild only. However, these definitions are generally intended for evaluating toxicity risk, while the intended use of the DDI metrics of this work is for evaluating comparative risk of treatment failure. Therefore, the inhibitor DDI metrics were adjusted to represent the equivalent of the induction exposure changes in the opposite direction, i.e., 25% decrease vs. 25% increase. These DDI flags then were incorporated into the final dataset to be used as covariates within the statistical analyses.

### 2.5. Statistical Analyses

Treatment failure rates for macrolides were calculated as a ratio of cases divided by the total number of events, with the success rate equal to 1 minus the failure rate. To estimate the relative odds of macrolide treatment failure, a conditional logistic regression model was fitted using the matched cases and controls. Optimization of the model fit was conducted using the Newton–Raphson algorithm. The overall model fit was evaluated using a likelihood ratio chi-square test (*p* < 0.05) at 4 degrees of freedom. Lastly, the odds ratio point estimates were determined using conditional maximum likelihood estimation and tested for significance using the Wald Chi-Squared test (*p* < 0.05) at 1 degree of freedom, along with the corresponding 95% Wald confidence intervals. 

## 3. Results

**Figure 1:**
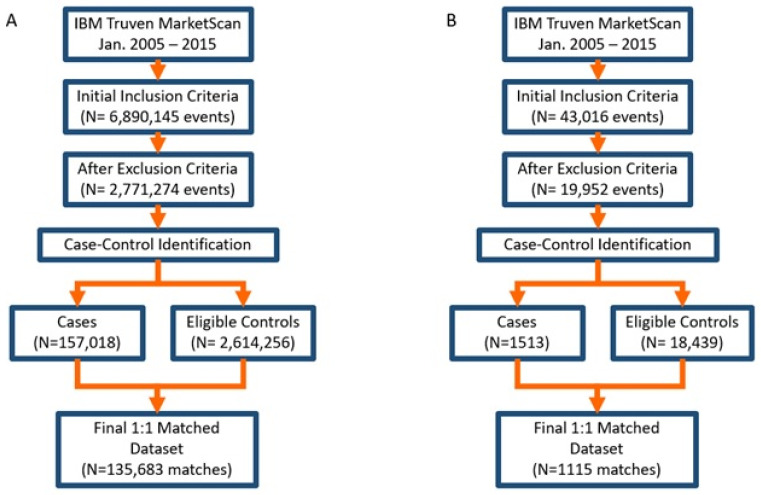
Summary of cohort generation for the common infections cohort (**A**) and bacterial pneumonia cohort (**B**) utilized in the study’s analyses.

A flowchart of the cohort generation steps and results is summarized in Figure 1 for both study cohorts. The final dataset for the acute infection analysis contained 135,683 case–control matches, i.e., 271,366 members. A significant portion of these patients received azithromycin (88.5%), with clarithromycin having the second highest proportion (11.2%) and erythromycin being the lowest proportion of use (0.03%). For the bacteria-specific pneumonia analysis, the final population contained 1115 case–control matches, with a significant portion receiving azithromycin (83.0%) and the rest receiving clarithromycin (17.0%). A complete summary of the final demographics of the study cohorts, in total and per macrolide, is summarized in Table 1 and Table 2.

Age was summarized as mean with (standard deviation). Note that cases and controls in this cohort were exact matched so demographics were the same for both groups.

Age was summarized as mean with (standard deviation) and NA indicates no availability within the study data,

Once the final study cohort was identified, the rates of treatment failure and success were calculated for the whole population, as well as per each macrolide. In general, the acute infection population had a treatment failure rate of 6%, with azithromycin being 5.6%, clarithromycin being 6.9% and erythromycin being 6.2%. In comparison, the bacteria-specific CAP population had a failure rate of 8%, with azithromycin being 7.6% and clarithromycin having a 10.6% failure rate. Full summaries of these rates are provided in Table 3 and Table 4. 

Finally, once the overall model fit was determined as statistically significant, the odds ratio estimates for each PK covariate were calculated for the total population, as well as limited to each macrolide. The acute infection results had statistically significant indications that patients have approximately 12% increased odds of macrolide failure when exposure is increased and approximately 35% increased odds of failure when exposure is decreased. While the bacteria-specific analysis seems to have similar trends to the acute infection analysis, no statistically significant results were observed for the calculated odds ratios. A full summary of the regression analysis and calculated odds ratios are provided in Table 5 and Table 6. 

## 4. Discussion

This work is the first scientific investigation for macrolide antibiotics that integrates a pharmacoepidemiologic approach to a clinical pharmacology hypothesis using PK exposure metrics as covariates. The regression results of this project indicated that risk of macrolide treatment failure is not significantly changed for any macrolide when exposure is only slightly altered, e.g., within weak inhibition or induction DDI scenarios. This notion is reflected in the fact that none of the calculated odds ratios were statistically significant for any of the mild AUC change groups. In comparison, the results do indicate that when macrolide AUC changes by greater than 50%, the risk of treatment failure can be significantly influenced. Specifically, based on the total population for the acute infection analysis, we see that the calculated odds ratio comparing failure risk of a patient with no macrolide AUC change with a matched patient with moderate AUC decrease is 1.37 [1.02,1.86]. In other words, the model reports a statistically significant approximation of a 37% increase in failure risk when a patient has a >50% decrease in macrolide AUC when compared to a similar patient without the exposure change. Interestingly, using the same method of interpretation as the AUC decrease, the results also show that a >50% macrolide AUC increase leads to a statistically significant approximation of a 12% increase in failure risk for the acute infection analysis. In comparison, the resulting odds ratio estimations of the bacteria-specific pneumonia analysis were found to be statistically insignificant, leading to a lack of confident conclusion on the treatment failure risks. 

With respect to failure rates measured in this work, it is somewhat unintuitive that the failure rates would be higher in the bacteria-specific pneumonia population compared to the common acute infection population. However, it can be assumed that the reason why these pneumonia patients were coded with a bacteria-specific pneumonia diagnosis code is that the treating physician had their sample tested prior to treating due to a suspicion of a treatment-resistant pathogen. Thus, patients who fall into this bacteria-specific category could have been predisposed to resistance and thus treatment failure to begin with, although this cannot be confirmed via medical claims alone.

The macrolide treatment failure criteria as an approximation for the emergence of macrolide resistance is an important concept for this work. While the treatment failure criteria have been well proven for accurately identifying macrolide therapy failure, the lack of clinical isolate data within the medical claims will inevitably lead to the possibility that the macrolide treatment failure was not due to macrolide resistance [20,21,22]. Failure could also be due to nonbacterial (i.e., viral) causes of infection rather than emerging differences in bacterial susceptibility only. However, given the extensiveness of the inclusion/exclusion criteria, exact matching of seven relevant variables, validated failure criteria and knowing the use of macrolides are the biggest drivers of resistance development, we believe that our results are at least in part representative of the emergence of resistance as a cause for treatment failure. 

While it is impossible to reliably determine the exact cause of treatment failure using claims data, we believe it is reasonable to assume that physicians appropriately diagnosed and treated their patients with macrolide antibiotics accordingly. To further account for this nonbacterial infection confounding, an additional analysis looking at only bacteria-specific pneumonia was performed. Bacteria-specific pneumonia was chosen for this second analysis due to the lack of ICD-9-CM coding that specifies bacteria-specific acute infections, i.e., sinusitis, bronchitis and AOM. Furthermore, macrolide treatment of pneumonia has been previously studied using claims data, which provides confidence in the methodological approach of this analysis [21,22]. Lastly, another possible source of bias with respect to this work is the use of a commercially insured population. The database used for this work only included patients covered under a commercial health plan, which may lead to unavoidable biases with respect to healthcare outcomes.

An important assumption of this work is one that exists in all studies involving pharmacy claims data: accurate drug exposure. For this work, we assume that all drugs contained in the patient’s pharmacy claims file were appropriately administered and consumed by the patient as described in the claim. While deemed reasonable to assume for research purposes, it is always possible that a patient fills their drug but never actually takes it. While there are some methods to measure drug adherence within pharmacy claims, most rely on multiple continuous fills for adherence estimation [29]. This reliance made such methods not possible to implement in this work due to the inclusion/exclusion criteria requiring no previous antibiotic use prior to the index date. In addition, due to frequent use of sample medications and low-cost generic programs in the U.S., it is also possible that some patients were exposed to additional DDI perpetrators but not observed using pharmacy claims [30,31]. Such biases would make our results conservative.

The results of the macrolide exposure influences on treatment failure are particularly interesting, especially for the notion of increased exposure leading to increased treatment failure. While it is intuitive that a greater than 50% decrease in macrolide AUC could increase the risk of treatment failure, i.e., not enough drug to kill bacteria, it is less intuitive that a greater than 50% AUC increase could also increase the risk of failure. However, when one considers the clinical pharmacology of the macrolides, especially azithromycin, such a possibility becomes more plausible. Evidence has been presented that increases in the length of time in which macrolide concentrations are below MIC_90_ levels significantly increases the risk of macrolide resistance [11]. Azithromycin has a significantly longer terminal half-life than that of erythromycin or clarithromycin, which contributes to longer sub-MIC levels [24]. Furthermore, studies have shown that azithromycin can be subjected to lysosomal trapping via molecular ionization, which can also contribute to extended periods of sub-MIC levels [12]. This referenced work also showed that the ionization trapping of azithromycin results in a slow drug release based on white blood cell turnover, significantly contributing to half-life length. With this in mind, the findings of this study support the hypothesis of these extended sub-MIC levels for azithromycin, as well as suggest that this period can be altered by interactions, which cause macrolide plasma exposure to increase. However, given the role of white blood cells during an immune response, one needs to be mindful of potential differences between healthy subjects and patients.

Two other important aspects of macrolide safety and efficacy that should be considered are the effect of obesity on treatment outcomes, as well as the proarrhythmic risk of macrolides. Due to the lipophilicity of macrolides, it could be expected that obese patients would have an increase in volume of distribution, which in turn can decrease plasma concentrations while increasing terminal half-life compared to a healthy body mass index (BMI) population. Such changes in PK would suggest that obese patients would experience macrolide treatment failure at a higher rate than healthy BMI patients; however, very few resources concerning macrolide therapy in obese populations are currently available [32]. At least one study did support this notion by showing that the rate of *H. pylori* eradication was significantly lower in obese patients compared to healthy BMI patients when receiving a triple therapy, which included clarithromycin [33]. However, more work is needed in this area of macrolide therapy. It is well known that oral use of macrolide antibiotics can increase the risk of tachyarrhythmia and sudden cardiac death [34]. Further, it has been shown that such risk occurs in a concentration-dependent matter, suggesting that exposure increases can contribute to higher proarrhythmic risk [35]. This is of relevance for both DDI situations, as well as long-term use of macrolides, particularly azithromycin, for treatment of GI motility issues and non-TB mycobacterium infections, where the effects of extended use of macrolides are not well-characterized for bacterial resistance or cardiotoxic risks [36]. 

While the results of this work themselves are not clinically actionable, they are critical for future investigations into the PK/PD of macrolide antibiotics specifically for optimizing their clinical utility while decreasing the risk of therapy failure. In fact, efforts are already underway by research groups to identify novel effective antibiotics with very short half-lives but formulated as extended-release products to avoid the emergence of resistance [37]. Using real-world outcomes data, the findings of this work provide insight into how macrolide exposure changes can potentially alter macrolide treatment success. This insight will be the foundation for future work to better understand the behavior of macrolide PK among various physiological conditions and how these behaviors can be utilized to optimize clinical value. Such future work includes physiologically based pharmacokinetic modeling to evaluate macrolide exposure and tissue distribution for various dosing scenarios and patient populations, as well as quantitative systems modeling to evaluate various infection scenarios. The overall goal is to optimize macrolide antibiotic utility while avoiding macrolide treatment failure and resistant bacteria development.

## 5. Conclusions

The integration of pharmacoepidemiology and clinical pharmacology provides a framework for utilizing real-world data to provide insight into mechanisms of macrolide failure and support future study development related to antibiotic treatment. In this work, we hypothesized and showed that changes in the pharmacokinetics of macrolide exposure, both increased or decreased, can potentially alter the risk of macrolide treatment failure. These results indicate that it is clinically important for antibiotic exposure to be precise and accurate, especially for long half-life antibiotics. Future work will focus on mechanistic modeling and clinical trial development for macrolide antibiotics.

## Figures and Tables

**Table 1:** Summary of the common acute infection study cohort demographic data in total, as well as broken down by macrolide used.

Total Population	Demographic Summary
Matched Pairs	135,683
Age (years)	40.6 (14.7)
Sex (M/F)	0.71
Azithromycin Only	
Matched Pairs	120,197
Age (years)	40.4 (14.9)
Sex (M/F)	0.70
Clarithromycin Only	
Matched Pairs	15,171
Age (years)	42.2 (13.4)
Sex (M/F)	0.81
Erythromycin Only	
Matched Pairs	315
Age (years)	42.9 (12.3)
Sex (M/F)	0.71

**Table 2:** Summary of the bacteria-specific CAP study cohort demographic data in total, as well as broken down by macrolide used.

	Demographic Summary	
Total Population	Case	Control
Matched Pairs	1115	1090
Age (years)	37.8 (17.1)	37.8 (17.0)
Sex (M/F)	0.93	0.93
Azithromycin Only		
Matched Pairs	926	920
Age (years)	37.4 (17.2)	37.5 (17.1)
Sex (M/F)	1	1
Clarithromycin Only		
Matched Pairs	189	170
Age (years)	39.4 (16.6)	39.4 (16.6)
Sex (M/F)	0.84	0.84
Erythromycin Only		
Matched Pairs	NA	NA
Age (years)	NA	NA
Sex (M/F)	NA	NA

NA indicates no cases were identified in the data.

**Table 3:** Summary of the macrolide treatment failure rates within the common acute infection analysis for the total study population and broken down by macrolide used. Data are presented as absolute number, as well as percentage.

	Treatment Failure	Treatment Success
Total Population	15,468 (5.7%)	255,898 (94.3%)
Azithromycin Only	13,462 (5.6%)	226,931 (94.4%)
Clarithromycin Only	2094 (6.9%)	28,248 (93.1%)
Erythromycin Only	39 (6.2%)	591 (93.8%)

**Table 4:** Summary of the macrolide treatment failure rates within the bacteria-specific pneumonia analysis for the total study population and broken down by macrolide used. Data are presented as absolute number, as well as percentage.

	Treatment Failure	Treatment Success
Total Population	172 (7.7%)	2058 (92.3%)
Azithromycin Only	141 (7.6%)	1711 (92.4%)
Clarithromycin Only	40 (10.6%)	338 (89.4%)
Erythromycin Only	NA	NA

NA indicates no cases were identified in the data.

**Table 5:** Summary of the calculated odds ratios and 95% confidence intervals for risk of treatment failure in the common acute infection analysis with respect to AUC change versus no AUC change due to DDI. Statistical significance of the odds ratio estimate was confirmed if the 95% confidence interval did not cross a value of 1.

**AUC Change Covariate**	**Odds Ratio for Treatment Failure**	**95% Confidence Interval**
Total Population		
Mild AUC Increase	0.99	0.92, 1.06
Moderate AUC Increase	1.12	1.08, 1.17
Mild AUC Decrease	0.56	0.30, 1.02
Moderate AUC Decrease	1.37	1.02, 1.86
Azithromycin Only		
Mild AUC Increase	0.98	0.91, 1.06
Moderate AUC Increase	1.12	1.08, 1.17
Mild AUC Decrease	0.64	0.34, 1.21
Moderate AUC Decrease	1.34	0.99, 1.85
Clarithromycin Only		
Mild AUC Increase	1.01	0.83, 1.25
Moderate AUC Increase	1.11	1.00, 1.23
Mild AUC Decrease	0.81	0.22, 3.03
Moderate AUC Decrease	1.64	0.68, 3.96
Erythromycin Only		
Mild AUC Increase	0.50	0.05, 5.51
Moderate AUC Increase	1.18	0.53, 2.64
Mild AUC Decrease	NA	NA
Moderate AUC Decrease	NA	NA

NA indicates no cases were identified in the data.

**Table 6:** Summary of the calculated odds ratios and 95% confidence intervals for risk of treatment failure in the bacteria-specific CAP analysis with respect to AUC change versus no AUC change due to DDI.

**AUC Change Covariate**	**Odds Ratio for Treatment Failure**	**95% Confidence Interval**
Total Population		
Mild AUC Increase	1.01	0.40, 2.53
Moderate AUC Increase	1.09	0.62, 1.92
Mild AUC Decrease	NA	NA
Moderate AUC Decrease	3.00	0.31, 28.8
Azithromycin Only		
Mild AUC Increase	1.01	0.38, 2.69
Moderate AUC Increase	1.21	0.66, 2.22
Mild AUC Decrease	NA	NA
Moderate AUC Decrease	***	***
Clarithromycin Only		
Mild AUC Increase	1.00	0.63, 15.9
Moderate AUC Increase	0.50	0.09, 2.73
Mild AUC Decrease	NA	NA
Moderate AUC Decrease	***	***
Erythromycin Only		
Mild AUC Increase	NA	NA
Moderate AUC Increase	NA	NA
Mild AUC Decrease	NA	NA
Moderate AUC Decrease	NA	NA

*** Indicates odds ratios could not be reliably estimated due to limited sample size. NA indicates no cases were identified in the data for the specified scenario.

## Data Availability

Not applicable.

## Supplementary Materials

The following supporting information can be downloaded at: https://www.mdpi.com/article/10.3390/pharmaceutics14040704/s1, Table S1. Bacterial-specific CAP ICD-9-CM Codes.
