# Supplementary material for: Macrolide Treatment Failure due to Drug–Drug Interactions: Real-World Evidence to Evaluate a Pharmacological Hypothesis
Source: Pharmaceutics. 2022 Mar 25;14(4):704. doi: 10.3390/pharmaceutics14040704 (PMC9031623; doi:10.3390/pharmaceutics14040704)
Supplement: Supplementary file 1 [file pharmaceutics-14-00704-s001.zip › pharmaceutics-1627823-supplementary.pdf]

pharmaceutics

Supplementary Materials: Macrolide Treatment Failure Due to Drug–Drug Interactions: Real-World Evidence to Evaluate a Pharmacological Hypothesis

Brian Cicali, Stephan Schmidt, Markus Zeitlinger and Joshua D. Brown

Table S1. Bacterial-specific CAP ICD-9-CM Codes.

ICD-9-CM	Textual Definition
481	Pneumococcal pneumonia [Streptococcus pneumoniae pneumonia]
482	Other bacterial pneumonia
482.0	Pneumonia due to Klebsiella pneumoniae
482.1	Pneumonia due to Pseudomonas
482.2	Pneumonia due to Hemophilus influenzae [H. influenzae]
482.3	Pneumonia due to streptococcus
482.30	Pneumonia due to Streptococcus, unspecified
482.31	Pneumonia due to Streptococcus, group A
482.32	Pneumonia due to Streptococcus, group B
482.39	Pneumonia due to other Streptococcus
482.4	Pneumonia due to staphylococcus
482.40	Pneumonia due to Staphylococcus, unspecified
482.41	Methicillin susceptible pneumonia due to Staphylococcus aureus
482.42	Methicillin resistant pneumonia due to Staphylococcus aureus
482.49	Other Staphylococcus pneumonia
482.8	Pneumonia due to other specified bacteria
482.81	Pneumonia due to anaerobes
482.82	Pneumonia due to escherichia coli [E. coli]
482.83	Pneumonia due to other gram-negative bacteria
482.84	Pneumonia due to Legionnaires' disease
482.89	Pneumonia due to other specified bacteria
482.9	Bacterial pneumonia, unspecified
